# Metastatic Colorectal Cancer Resembling Severe Preeclampsia in Pregnancy

**DOI:** 10.1155/2015/487824

**Published:** 2015-12-07

**Authors:** Raminder Kaur Khangura, Charanpreet Kaur Khangura, Anagha Desai, Gregory Goyert, Roopina Sangha

**Affiliations:** ^1^Department of Obstetrics and Gynecology, Henry Ford Hospital, Detroit, MI, USA; ^2^Nova Southeastern University College of Osteopathic Medicine, Davie, FL, USA

## Abstract

Although colorectal cancer (CRC) is the third most common cancer in women, it is a rare malignancy in pregnancy. Symptoms of CRC such as fatigue, malaise, nausea, vomiting, rectal bleeding, anemia, altered bowel habits, and abdominal mass are often considered typical symptoms of pregnancy. Many cases of CRC are diagnosed in advanced stages due to missed warning signs of CRC, which may be misinterpreted as normal symptoms related to pregnancy. This report reviews 2 cases of CRC diagnosed within a 4-month interval at our institution. Both cases were initially thought to be atypical presentations of preeclampsia. Prenatal history, hospital course, and postpartum course were reviewed for both patients. CRC is often diagnosed at advanced stages in pregnancy. Common physiological symptoms of pregnancy should be scrutinized carefully and worked up appropriately, especially if symptoms remain persistent or increase in intensity or severity.

## 1. Introduction

Colorectal cancer (CRC) has a reported incidence of 0.002% in pregnancy [1]. The mean age of diagnosis of CRC in pregnancy is 31 years old [1]. Delayed childbearing and increased maternal age are factors that may lead to an increased incidence of CRC complicating pregnancy [2].

## 2. Case Presentation

**Case 1 .**

A 34-year-old G4P2012 with a known medical history of neurofibromatosis presented at 35-week gestation after a fall with complaints of nausea, vomiting, and right upper quadrant pain. Her nausea and vomiting improved with hydration. The right upper quadrant pain was attributed to musculoskeletal pain due to the fall. Laboratory evaluation demonstrated an isolated, elevated aspartate aminotransferase of 117 U/L and she was discharged home after she tolerated diet with subjective improvement in symptoms.

The patient returned 5 days later due to recurrent right upper quadrant pain, headache, and contractions. The patient's contractions improved after terbutaline and pain relief was achieved with fentanyl. Fetal status remained reassuring. HELLP laboratory studies were drawn and showed the aspartate aminotransferase to be elevated to 166 U/L and lactate dehydrogenase (LDH) elevated to 1546 *μ*kat/L. The patient's blood pressures remained normotensive; however, an atypical presentation of preeclampsia was considered given the elevated liver enzymes and symptomatology of preeclampsia including headache and right upper quadrant pain. A timed 24-hour urine protein was performed to rule out atypical presentation of preeclampsia, which showed 340 mg of protein.

An abdominal ultrasound was obtained due to persistent right upper quadrant pain and showed hepatomegaly with the right hepatic lobe measuring 21 cm diagonally with multiple confluent echogenic masses visualized within the liver. The elevation of LDH was attributed to infiltration of the liver by hepatic lesions, contributing to hemolysis. Magnetic resonance imaging (MRI) was recommended to evaluate the ultrasound findings.

On day 2 of hospitalization, the patient had diarrhea and continued to have sharp right upper quadrant pain. MRI studies revealed near complete replacement of the liver by innumerable hepatic masses, some of which demonstrated central cystic changes which were thought to possibly represent neurofibromas or other tumors associated with neurofibromatosis given the patient's history. Metastatic disease was considered unlikely due to the lack of a T2 hyperintense signal.

**Figure 1:**
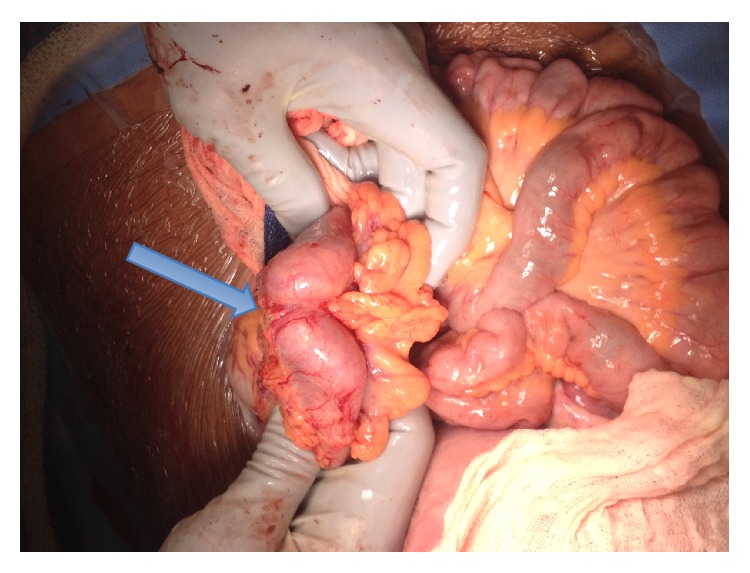
Transverse colonic mass at time of cesarean section.

**Figure 2:**
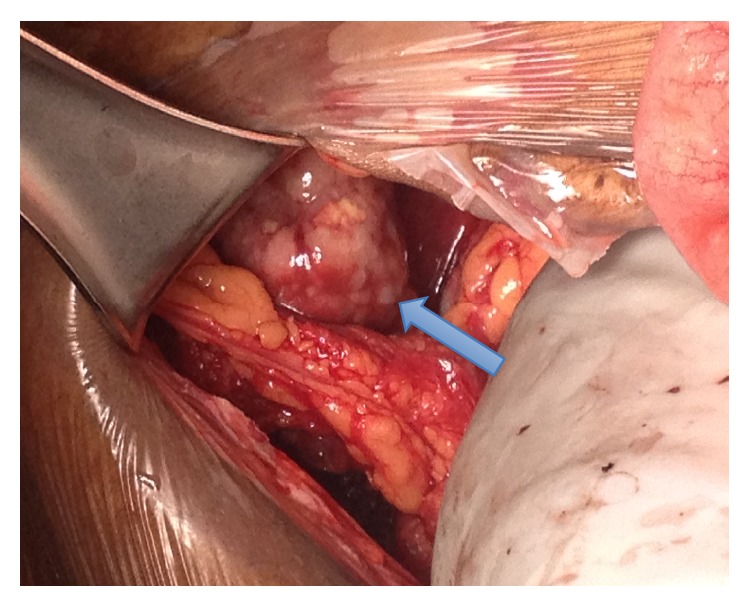
Metastatic disease noted on the liver.

Ultimately, the decision was made to proceed to delivery via cesarean section at 36-week gestation due to persistent right upper quadrant pain and worsening of bilirubin and LDH. A multidisciplinary operating team was organized with gynecologic oncology and transplant surgery on standby for exploration of the abdomen and evaluation of the liver after delivery of the infant. At the time of surgery, a 4–6 cm transverse colon mass was identified with extensive tumor burden in both liver lobes (Figures 1 and 2). Two core needle biopsies and 1 excisional biopsy were obtained. Enlarged para-aortic lymph nodes were also appreciated. Intraoperative consultation was made by surgical oncology and the plan was to avoid removal of the transverse colon mass, which would not benefit the patient from the disease process and would put her at risk of an anastomotic leak. Instead, a diverting loop colostomy was performed by general surgery.

The postoperative course was uneventful. Hematology oncology was consulted and carcinoembryonic antigen (CEA) was obtained, which demonstrated elevation at 2131.5 *μ*g/L. The pathology report confirmed metastatic carcinoma of the colon. A computed tomography (CT) scan was obtained of the chest, abdomen, and pelvis and was negative except for the known masses of the liver and colon. The patient was found to have stage IV colon cancer. Genetic testing was obtained to evaluate the patient for Lynch syndrome and other mutations contribute to premature colon cancer. Gene mutation analysis showed positive Kras with point mutation indicated by the nucleotide change in codon 12 and negative BRAF mutation. Microsatellite instability testing showed normal expression of MLH1, MSH2, MSH6, and PMS2 proteins without any altered markers. The patient initiated FOLFOX 4 weeks after her operation.

**Case 2 .**

A 44-year-old gravida 3 para 2 presented to labor and delivery triage due to severe contractions. Her prenatal course was only significant for gestational diabetes, which was controlled with glyburide, and back pain starting at 35 weeks, for which the patient took acetaminophen with Codeine number 3 on occasion to relieve her discomfort. The patient was scheduled for repeat cesarean section at 39 weeks due to a prior history of 2 cesarean sections. The patient was found to be in labor and underwent an uncomplicated repeat cesarean section with bilateral tubal ligation.

On postoperative day 2, the patient was noted to be hypertensive in the range of 147–161/83–101 mmHg, which was elevated from her baseline blood pressures. The patient also had complaints of headache, back pain, nausea, and vomiting. Postpartum preeclampsia was considered the likely diagnosis. Laboratory studies were drawn and showed the following: aminotransferase elevated at 89 U/L, alanine transaminase at 26 U/L, creatinine at 55.6 *μ*mol/L, and LDH elevated at 22.3 *μ*kat/L. The patient was transferred back to labor and delivery and started on magnesium sulfate for postpartum preeclampsia and also labetalol 200 mg three times daily.

The patient's blood pressures stabilized on antihypertensive medications, which were discontinued after twenty-four hours because of normalization of blood pressures. Her laboratory findings remained abnormal for an elevated aspartate aminotransferase ranging between 73 and 110 U/L and also an elevated LDH between 23 and 33 *μ*kat/L. The patient later spiked temperatures up to 39°C. She was started on broad-spectrum antibiotics for postpartum endometritis. Blood cultures and urine cultures were obtained. The urine culture returned as positive for* Pseudomonas aeruginosa*, and the blood cultures were positive for* Klebsiella pneumoniae*. Broad-spectrum antibiotics were discontinued and the patient was started on cefepime. Per infectious disease consult, a renal ultrasound was also obtained to evaluate for pyelonephritis and nephrolithiasis.

Renal ultrasound showed normal kidneys and bladder and revealed a large solid hepatic mass with a central cystic component measuring 9.4 × 8.1 × 14.0 cm. A CT scan revealed large confluent hypodense masses involving the right and left hepatic lobes and enlarged retroperitoneal and mesenteric lymph nodes. A persistently thickened segment of the distal ascending colon/proximal pelvic flexure was also noted. There were no findings of metastatic process in the chest. Hepatology was consulted and further laboratory studies were obtained including cancer antigen (CA) 125: 67 kU/L; alpha-fetoprotein: 34.2; CEA: 552.3 mcg/L; and CA 19-9 at 23608 kU/L. Further work-up was recommended to differentiate between breast, ovarian, pancreatic, or colonic primary cancers. Screening mammography was within normal limits. A CT guided liver biopsy ultimately confirmed adenocarcinoma of colonic origin. A colonoscopy was performed, which showed a friable mass in the ascending colon. A biopsy of the mass also showed adenocarcinoma. Kras was ordered and was mutated. Diagnosis of stage IV colon cancer was confirmed. The patient started palliative chemotherapy with FOLFOX after being discharged from the hospital.

## 3. Discussion

CRC is one of the most common types of cancer in women. While the highest incidence of CRC occurs in patients aged 50 years or older, 3% of patients with CRC are younger than 40 years of age and it is the seventh most common type of cancer in pregnancy with a reported incidence of 0.002%. [1–3]. The median age in reported cases of diagnosis was 31 years, with most cases being reported in very advanced stages due to diagnostic challenges [1, 3]. The presenting signs and symptoms of CRC include nausea, vomiting, abdominal pain, altered bowel movements, rectal bleeding, and anemia which can be commonly found in pregnancy. Due to this reason, many of these signs and symptoms are typically overlooked and an appropriate work-up is not initiated until the cancer is already in advanced stages [1–5]. In our second case, the diagnosis of colon cancer was an incidental finding while evaluating the kidneys due to a urinary tract infection and bacteremia with the patient only complaining of nausea, vomiting, and back pain during her third trimester.

Signs and symptoms of CRC in pregnancy such as persistent rectal bleeding, nausea and vomiting, constipation, and weight loss should be worked up. Hemorrhoids, the common cause of rectal bleeding, should be visualized and rectal examination should be performed when a patient presents with a complaint of rectal bleeding. In addition, if a patient starts to lose weight while pregnant, she should be evaluated for maternal and fetal etiologies. Persistent nausea and vomiting in pregnancy should be evaluated further, especially in the third trimester [1–6].

Treatment goals of the mother take into account the gestational age of the fetus, fetal lung maturity, cancer stage, and the need for emergent or elective surgery. The goal is to start treatment for the mother as early as possible. If the cancer is detected at a more advanced gestational age, delivery of the viable infant can occur at 32 weeks if the lungs are matured [6]. Treatment modalities may include surgery, radiation therapy, and chemotherapy depending on the stage of the cancer [6].

Pregnant women with colorectal cancer generally have a poor prognosis due to the late diagnosis of the disease. In a review of 42 patients with colorectal cancer in the literature, Chan et al. noted that 23 (56%) of these patients died by the time the cases were reported in the literature [2, 7]. Most died within fifteen months of diagnosis and the median survival for the group was less than 5 months. One patient survived 3.5 years after bowel resection with multiple recurrences. No patient with colorectal cancer in pregnancy in the literature has survived longer than five years [2, 7].

In conclusion, CRC is an aggressive cancer that is rarely found in pregnancy. However, when CRC is found in pregnancy it is usually diagnosed in the late stages. Early diagnosis improves survival and treatment outcomes. Thorough physical examination should be performed on patients presenting in the early first trimester before the gravid uterus becomes prominent with special attention given to the gastrointestinal evaluation, which should include the evaluation of the size of the liver and a rectal examination to screen for masses. Common physiological symptoms of pregnancy should be scrutinized carefully and worked up appropriately, especially if symptoms remain persistent or increase in intensity or severity. A thorough evaluation of abnormal laboratory values and consideration of nonobstetric disease processes is important to optimize patient outcomes in complicated obstetric patients.
